# PD-L1 is an activation-independent marker of brown adipocytes

**DOI:** 10.1038/s41467-017-00799-8

**Published:** 2017-09-21

**Authors:** Jessica R. Ingram, Michael Dougan, Mohammad Rashidian, Marko Knoll, Edmund J. Keliher, Sarah Garrett, Scott Garforth, Olga S. Blomberg, Camilo Espinosa, Atul Bhan, Steven C. Almo, Ralph Weissleder, Harvey Lodish, Stephanie K. Dougan, Hidde L. Ploegh

**Affiliations:** 1 Whitehead Institute for Biomedical Research, Cambridge, MA 02142 USA; 2 Department of Cancer Immunology and Virology, Dana-Farber Cancer Institute, Boston, MA 02115 USA; 3 Division of Gastroenterology, Massachusetts General Hospital, Boston, MA 02114 USA; 4 Center for Systems Biology, Massachusetts General Hospital, Boston, MA 02114 USA; 5 Department of Radiology, Massachusetts General Hospital, Boston, MA 02114 USA; 6 Department of Biochemistry, Albert Einstein College of Medicine, 1300 Morris Park Avenue, Bronx, NY 10461 USA; 7 Department of Pathology, Massachusetts General Hospital, Boston, MA 02114 USA; 8 Department of Systems Biology, Harvard Medical School, Boston, MA 02115 USA; 9 Department of Biology, Massachusetts Institute of Technology, Cambridge, MA 02142 USA; 10 Department of Microbiology and Immunology, Harvard Medical School, Boston, MA 02115 USA

## Abstract

Programmed death ligand 1 (PD-L1) is expressed on a number of immune and cancer cells, where it can downregulate antitumor immune responses. Its expression has been linked to metabolic changes in these cells. Here we develop a radiolabeled camelid single-domain antibody (anti-PD-L1 VHH) to track PD-L1 expression by immuno-positron emission tomography (PET). PET-CT imaging shows a robust and specific PD-L1 signal in brown adipose tissue (BAT). We confirm expression of PD-L1 on brown adipocytes and demonstrate that signal intensity does not change in response to cold exposure or β-adrenergic activation. This is the first robust method of visualizing murine brown fat independent of its activation state.

## Abstract (web summary)

Current approaches to visualise brown adipose tissue (BAT) rely primarily on markers that reflect its metabolic activity. Here, the authors show that PD-L1 is expressed on brown adipocytes, does not change upon BAT activation, and that BAT volume in mice can be measured by PET-CT with a radiolabeled anti-PD-L1 antibody.

## Introduction

Monoclonal antibodies that target the immunological checkpoints PD-1 (programmed cell death protein 1; CD279) and PD-L1 (programmed death ligand 1; CD274) have proven successful in the treatment of multiple cancers, notably metastatic melanoma, where PD-1 blockade is now part of the standard of care^1–4^. PD-L1 shows broad but low expression on myeloid cells and in other tissue types, but in response to interferon-γ (IFNγ), its expression increases. A wide range of human and murine malignancies express PD-L1 constitutively or inducibly^5–7^. Despite robust expression in the tumor microenvironment and in the setting of chronic viral infections, expression of PD-L1 in naive mice is low, and mice lacking PD-L1 show only modest immunologic aberrations^8^.

Despite the impressive gains in immunotherapy for cancer, heterogeneous outcomes necessitate new methods to monitor and predict patient responses. A method that comprehensively monitors PD-L1 expression could be of diagnostic value, and may help resolve lingering questions about the role of PD-L1 expression in checkpoint blockade responses^9–11^. To this end, we developed camelid single-domain antibodies, also known as VHHs, against immune surface proteins to monitor inflammation in the tumor microenvironment by immuno-positron emission tomography-computed tomography (PET-CT)^12^. We sought to extend our method to imaging lower abundance immune receptors, and chose PD-L1 as both a clinically relevant target and a protein with weak expression in naive animals^4, 9–11^. In the course of these experiments, we identified brown adipocytes as the major source of surface-disposed PD-L1 expression in naive mice.

Activated brown adipose tissue (BAT) increases body temperature and energy expenditure in infants and hibernating animals^13^. In brown adipocytes, the generation of ATP from the breakdown of glucose and fatty acids is interrupted by the expression of Ucp1 (Uncoupling protein 1) in the inner mitochondrial membrane, where it dissipates the proton gradient established by the electron transport chain with concomitant release of heat^14^. Ucp1 expression is up-regulated by cold exposure and subsequent signaling through β-adrenoreceptors^14, 15^ While it was previously believed that adult mammals lack BAT, imaging with the glucose analog 2-^18^F-fluorodeoxyglucose (^18^F-FDG) by positron emission tomography (PET) shows that adult humans have small residual BAT stores^16–18^. BAT is typically identified with functional markers that monitor BAT activity, using traceable metabolites like ^18^F-FDG, but there are currently no means to visualize non-activated BAT, although tissue can be identified by histological means in the absence of ^18^F-FDG uptake^19^. Unlike metabolite-based imaging reagents, the ability to visualize PD-L1 expression on brown adipocytes is independent of temperature exposure or β-adrenergic signaling, and shows robust staining of BAT deposits not visible by other non-invasive methods. Our studies thus provide a new tool to screen for therapeutic interventions of BAT function in metabolic disorders. Imaging of BAT will be essential if we are to harness its biology for the treatment of obesity, type 2 diabetes, and other metabolic disorders^20^.

## Results

### Generation of a single-domain antibody against mouse PD-L1

We immunized an alpaca with the purified ectodomain of mouse PD-L1, leading to the isolation by phage display of two single-domain antibodies (VHHs), termed B3 and A12, both of which bind specifically to PD-L1 with overlapping binding epitopes and estimated affinities in the low nM range (Supplementary Fig. 1a–f)^21–23^. We mapped the epitope recognized by B3 using a panel of HEK 293 derivatives transfected with constructs specifying single amino acid substitutions in PD-L1. When we mapped the mutations that abolish binding of B3 to PD-L1 onto the known structure of PD-L1, they clustered in and around the site known to interact with its ligand, programmed cell death protein 1 (PD-1) (Supplementary Fig. 2A; Supplementary Table 1), suggesting that B3 could block interactions between PD-L1 and PD-1. *In vitro* competition experiments confirmed that B3 competes for binding with both PD-1 and the alternative ligand B7-1 (CD80) for PD-L1 binding, with a potency similar to that of the commercial monoclonal antibody (Supplementary Fig. S2B)^5^.

### PET/CT with ^18^F- B3 reveals PD-L1 on brown adipose tissue

**Fig. 1:**
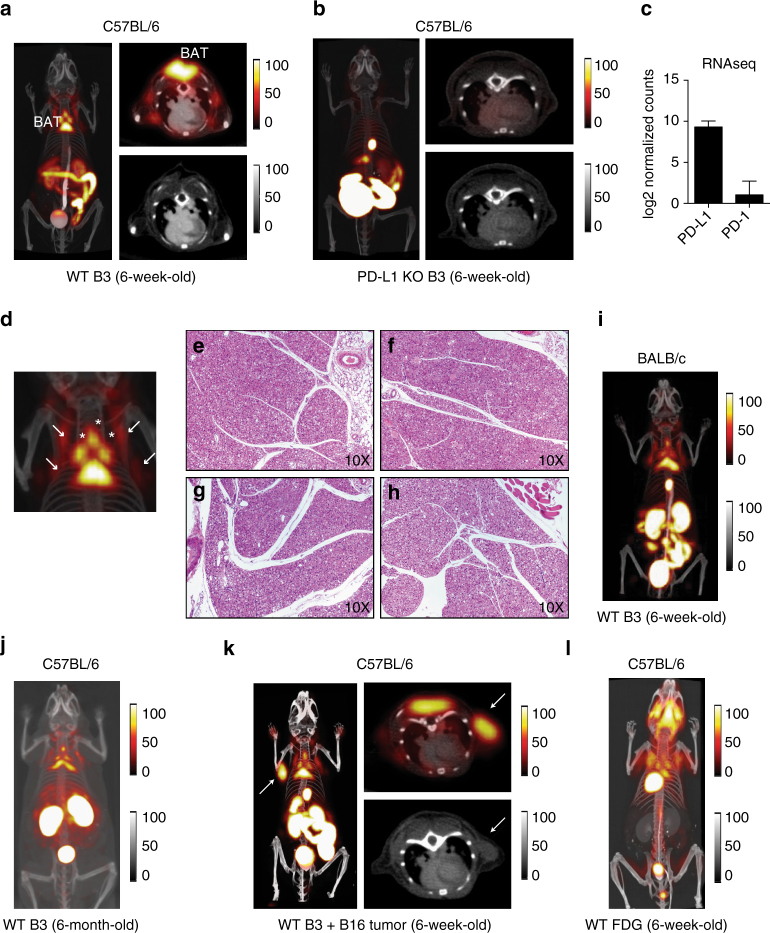
PD-L1 immuno-PET reveals expression in brown adipose tissue. **a**, **b** PET-CT 3D projection images of 6-week-old C57BL/6 mice (*left panel*), PET-CT axial slice through the brown adipose tissue (BAT) (*top right panel*) with companion CT axial image (*bottom right panel*). **a** ^18^F-B3 image from a WT mouse. **b** ^18^F-B3 image from a PD-L1 knockout (KO). **c** PD-L1 and PD-1 transcript levels measured by RNAseq in WT BAT. **d** Magnification of 3D projection of thorax from **a**. *Arrows* show BAT deposits. **f**–**h** H&E stained tissue sections 10× magnification. **e**, **f** Principal interscapular BAT deposit from WT **e** and PD-L1 KO **f** mice. **g**, **h** small parathoracic BAT deposits from WT **g** and PD-L1 KO **h** mice. **i**, **j** 18F-B3 PET-CT 3D projection image of a 6-week-old BALB/c WT mouse **i** and 6-month-old C57BL/6 WT mouse **j**. **k** 18F- B3 image from a WT mouse injected with subcutaneous B16 melanoma (*arrow*). **l** 18F-FDG image of 5-week-old C57BL/6 mouse. Images are all window-leveled to the same intensity. Three biological replicates were performed for each experiment. *Error bars* indicate standard error of mean (SEM)

PET-CT imaging with ^18^F-labeled B3 showed a robust signal two hours after injection in interscapular brown adipose tissue (BAT) in wild-type (WT), but not PD-L1 knockout (KO), mice (Fig. 1a, b; Supplementary Fig. 1a). Labeling of B3 with ^18^F or ^64^Cu produced equivalent results in terms of tissue distribution, with non-specific staining in the lumen of the gut and in kidney, a trait common to all VHHs used in PET imaging (Supplementary Fig. 3b). The B3 VHH binds PD-L1 in vivo, and thus BAT expresses PD-L1. The presence of PD-L1 is evident not only in the intrascapular region, but covers the distribution of all brown fat in the thorax, with small deposits below the scapulae and in elongated structures parallel to the spinal column, presumably equivalent to the paravertebral BAT deposits seen in humans^13^ (Fig. 1a). The presence of PD-L1 within BAT was evident at the RNA level as well (Fig. 1c). Histology of each of these deposits in both WT and PD-L1 KO mice confirmed their identity as BAT, with no obvious morphological differences between the two strains (Fig. 1d–h). PET imaging with full-sized antibodies fails to reveal any of these smaller structures and produces images of far lower resolution, while they are clearly distinguishable with our method^11^. Surgical removal of the interscapular brown fat from mice injected with radiolabeled versions of B3 and A12 followed by scintillation counting of the isolated organ further demonstrated specificity of the PET signal for BAT (Supplementary Fig. 3B). While both PD-L1-specific VHHs B3 and A12 showed comparable tissue distributions, a control VHH labeled with ^64^Cu showed no accumulation of label in BAT (Supplementary Fig. 3C).

We next examined whether PD-L1 expression was a general property of BAT across strains, and remained present as mice age. PD-L1 as detected by immuno-PET was present in BALB/c mice with a distribution similar to the C57BL/6 animals (Fig. 1i). Older animals maintained expression of PD-L1, with BAT deposits clearly visible by anti-PD-L1 PET in 6-month-old mice, The observed distribution is consistent with the expected decrease in BAT mass, as observed by other methods (Fig. 1j)^24, 25^. While we saw only a faint anti-PD-L1 signal in lymphoid tissue, mice implanted with the B16 melanoma showed strong, tumor-specific staining comparable to that seen in BAT (Fig. 1k; Supplementary Movie 1), consistent with known positivity of B16 for PD-L1. BAT is therefore the major site of PD-L1 expression in the naive mouse.

We imaged C57BL/6 mice with ^18^F-FDG to compare visualization of BAT with our anti-PD-L1 imaging method. ^18^F-FDG imaging showed fully overlapping BAT structures as seen with anti-PD-L1 immuno-PET, although at considerably lower resolution (Fig. 1l)^26^. PD-L1 immuno-PET thus represents an advance not only in imaging low abundance immune receptors, but also offers improved resolution for the identification of small brown fat deposits in the mouse.

### PD-L1 is expressed on brown adipocytes

**Fig. 2:**
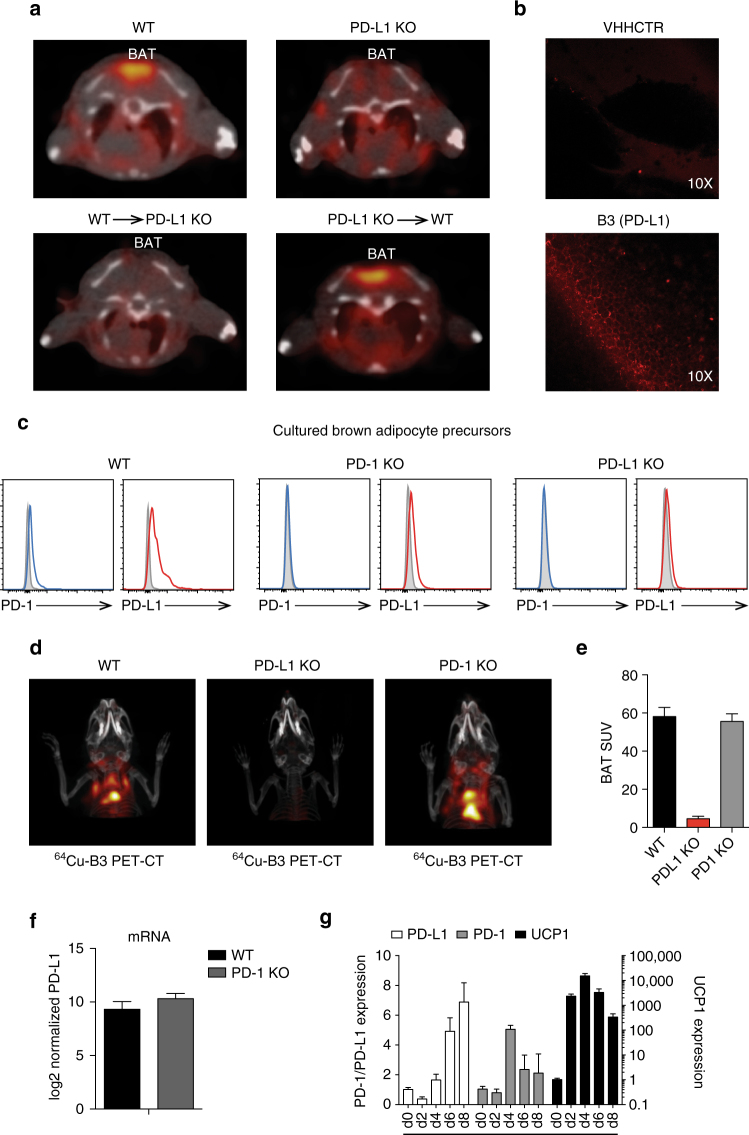
Non-hematopoietic cells in BAT express PD-L1. **a** PET-CT axial slice through BAT imaged with ^64^Cu-B3. (*Top panels*) WT (*left*) and PD-L1 KO (*right*) mice. (*Bottom panels*) Bone marrow chimeras; WT bone marrow into PD-L1 KO mice (*left*), and PD-L1 KO bone marrow into WT mice (*right*). *N* = 3 per chimera. **b** Confocal microscopy on brown fat resected from WT mice injected with Alexa647-labeled B3 or a non-specific control VHH. **c** Flow cytometry using the indicated antibody on cultured pre-adipocytes isolated from WT, PD-L1 KO, and PD-1 KO BAT. **d** Mice were imaged by PET-CT two hours after injection with ^64^Cu-B3. A 3D projection image comparing the torso and head of imaged WT, PD-L1 KO, and PD-1 KO mice is shown. **e** Quantification of background subtracted BAT signal from C57BL/6 mice analyzed as in **d**. Two mice were analyzed per group. **f** PD-L1 transcript levels measured by RNAseq in WT and PD-1 KO BAT **g** QPCR for PD-L1, PD-1 and Ucp1 transcripts on in vitro differentiated brown adipocytes. *X* axis indicates the day of culture after the induction differentiation. Each result is representative of at least two independent experiments. *Error bars* indicate SEM

Given the intensity of signal in the BAT compared to that in primary immune organs, we asked whether brown adipocytes themselves expressed PD-L1. We established WT into PD-L1−/− and PD-L1−/− into WT bone marrow (BM) chimeras and imaged them 7–8 weeks after transplant with radiolabeled B3 to determine whether PD-L1 was expressed on non-hematopoietic cells in BAT. At this time point, chimerism in the spleen is complete for both lymphoid and myeloid cells (data not shown). We saw a strong PET-CT signal in BAT from irradiated WT mice engrafted with PD-L1 KO bone marrow, a signal absent from PD-L1 KO recipients that received WT bone marrow (Fig. 2a). Consistent with this finding, WT mice injected with Alexa647-labeled B3 showed staining of BAT cells with adipocyte morphology by confocal microscopy; such signal was absent in animals injected with a labeled control VHH of irrelevant specificity (Fig. 2b). These results establish that cells of hematopoietic origin do not measurably contribute to the PD-L1 signal seen in BAT.

We next harvested pre-adipocytes from the BAT of three-week old WT mice^27^. Low levels of expression of PD-L1 and PD-1 on pre-adipocytes were evident by flow cytometry (Fig. 2c). Although PD-L1 expression appears reduced in cultured pre-adipocytes from PD-1 KO mice, PD-L1 expression in the mature BAT from PD-1 KO mice was maintained at the transcript level, and could be visualized by PET/CT (Fig. 2d–f). Analysis by quantitative PCR of in vitro differentiated brown adipocytes showed that levels of PD-L1 and PD-1 mRNA increase in the course of differentiation. Both genes are thus expressed in brown adipocytes and their expression is regulated during brown adipocyte development in vitro (Fig. 2g).

**Fig. 3:**
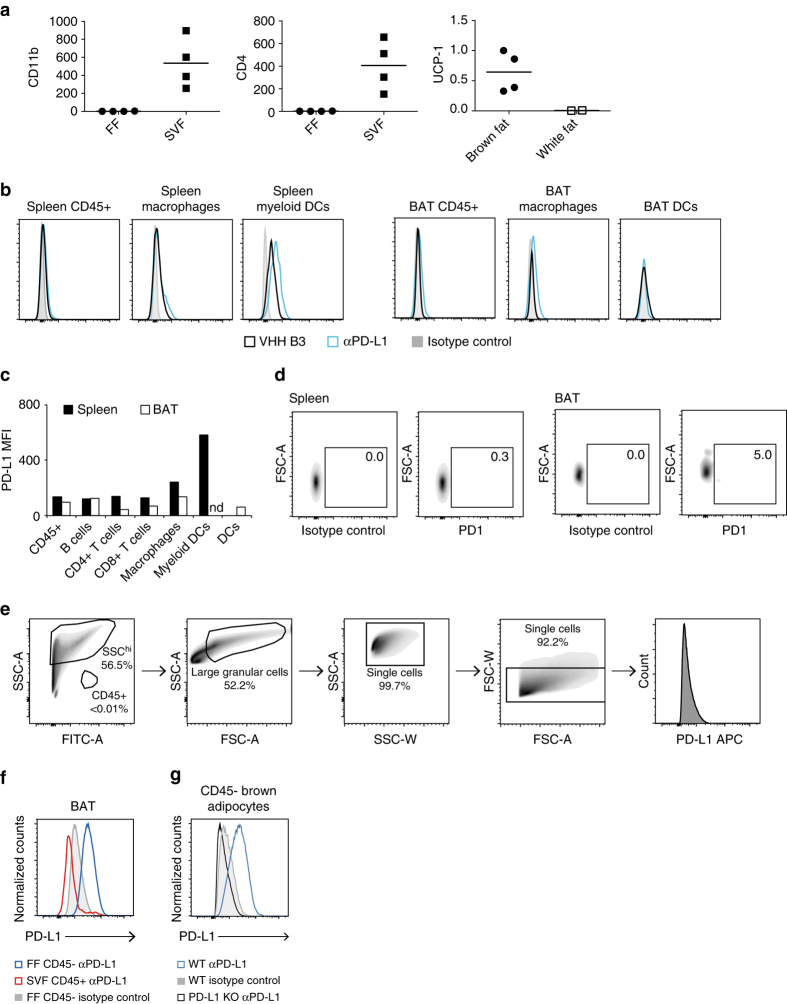
PD-L1 is expressed on brown adipocytes. **a** QPCR for the immune genes CD11b and CD4 in the floating fraction (FF) (*left panel*) and stromovascular fraction (SVF) (*middle panel*), and the brown fat gene UCP-1 in the FF from isolated brown fat and white fat. Y-axis shows relative expression normalized to 18S. **b** PD-L1 flow cytometry using αPD-L1 or B3 as indicated on CD45 + populations extracted from BAT SVF or spleens of WT mice. PD-L1 is only clearly detectable in splenic macrophages and myeloid DCs. *Y* axis is normalized cell counts. *X* axis shows MFI for each antibody or VHH as indicated in the figure legend. **c** Quantification of the data in **b** including B cell and T cell populations. **d** PD-1 flow cytometry on CD45 + cells from WT spleen and BAT SVF. **e** The lipid fraction was analyzed by flow cytometry and brown adipocytes were identified as CD45− large granular cells using the gating strategy shown on a representative sample from a WT mouse stained with isotype control antibody. The great majority of the small granular counts are from free lipid derived from ruptured adipocytes. **f** Flow cytometry using anti-PD-L1 or isotype control antibody on FF CD45− brown adipocytes isolated as in **e** compared to CD45 + cells from the SVF. **g** Flow cytometry on brown adipocytes as in **f** isolated from FF WT and PD-L1 KO mice. Each result is representative of at least two independent experiments with six pooled BAT samples per experiment

To show that brown adipocytes themselves express PD-L1 by an independent method, we analyzed them by flow cytometry. Isolation of brown adipocytes from BAT poses a challenge because of their inherent fragility^28^. We harvested brown adipocytes from the floating fraction (FF) of collagenase-digested BAT. This isolated fraction contained high levels of Ucp1 RNA when compared to floating cells isolated from subcutaneous white adipose tissue (scWAT), and was negative for hematopoietic markers when compared to the stromal vascular fraction (SVF), confirming the purity of mature brown adipocytes (Fig. 3a). As predicted by imaging of the bone marrow chimeras, flow cytometry on CD45^+^ hematopoietic cells isolated from the SVF showed minimal PD-L1 expression on all cell types examined, with levels far below that of PD-L1 + myeloid DCs in the spleen, a tissue where PD-L1 is barely detectable by immuno-PET (Fig. 3b, c). The CD45^+^ cells in the SVF also showed minimal expression of PD-1 in wild-type mice (Fig. 3d). Brown adipocytes were gated from the FF and analyzed separately for PD-L1 expression (Fig. 3e). As expected, the PD-L1 signal from the brown adipocytes was considerably higher than that observed in the CD45 + fraction (Fig. 3f), with staining altogether absent on brown adipocytes from PD-L1 KO mice (Fig. 3g). Taken together, these data establish the expression of PD-L1 on brown adipocytes.

### PD-L1 is an activation-independent marker of brown fat

**Fig. 4:**
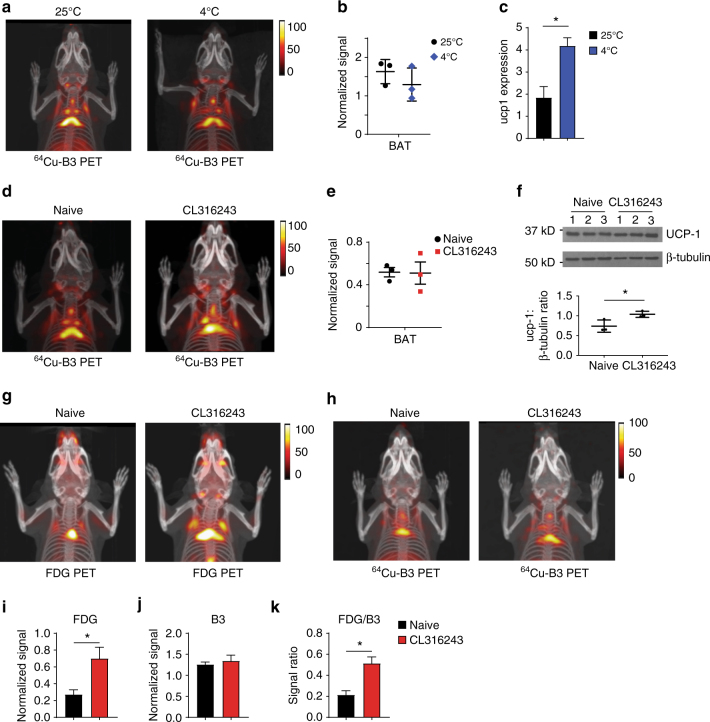
PD-L1 is an activation-independent marker of BAT. **a** ^64^Cu-B3 PET-CT 3D projection image of age and sex matched C57BL/6 mice held at room temperature (left panel) or held at 4C (right panel) for 90 min prior to injection of radiolabeled B3. Imaging was performed 60 min after ^64^Cu-B3 injection. **b** PET signal of BAT normalized to spleen from matched animals treated and imaged as in **a**. **c** Ucp-1 qPCR on RNA isolated from BAT excised from mice included in **b**. **d** ^64^Cu-B3 PET-CT 3D projection image acquired as in **a** of age and sex matched C57BL/6 mice treated with 10 mg/kg of the β-adrenergic agonist CL316243 given i.p. 45 min before injection of radiolabeled B3 (*right panel*) or left untreated (*left panel*, naive). **e** PET signal of BAT normalized to spleen and muscle from matched animals treated and imaged as in **d**. **f** Immunoblot for UCP-1 (top panel) on lystates from BAT excised from the mice included in **d** with quantification of normalized UCP-1 signal intensity (*bottom panel*) Numbers indicate biological replicates. **g**, **h** ^18^F-FDG **g** or ^64^Cu-B3 **h** PET-CT 3D projection image acquired on naive or CL316243-treated mice as in **c**. The mice in **g**, **h** are identical but treated and imaged 24 h apart (^18^F-FDG followed by ^64^Cu-B3) to allow for ^18^F radiodecay. **i**-**k** Matched animals were treated and imaged as **g**, **h** and PET signal was normalized and quanitifed. **i** ^18^F-FDG signal of BAT normalized to muscle and liver. **j** ^64^Cu-B3 signal of BAT normalized to spleen. **k** signal ratio of ^18^F-FDG to ^64^Cu-B3 for each mouse represented in **i**, **j**. *Error bars* throughout show SEM. Statistical significance was determined by a Student’s *t*-test. Three biological replicates were performed for each condition in each experiment

^18^F-FDG is currently the preferred method to image BAT non-invasively and does so at low resolution when compared to anti-PD-L1 immuno-PET. The identification of brown fat using ^18^F-FDG and other metabolite-based imaging reagents (e.g.,^11^C-acetate, 14(R,S)-^18^F-fluoro-6-thia-heptadecanoic acid) is linked to its metabolic activity, specifically the presence and activity of the glucose transporters responsible for uptake of ^18^F-FDG^29^. Cold exposure or treatment with β-adrenoreceptor agonists can modulate ^18^F-FDG uptake but cannot visualize BAT mass per se. Imaging of total BAT volume, independent of its metabolic activity has not been possible until now. This is a serious limitation, when screening for metabolic interventions considered for the treatment of obesity, type 2 diabetes and other metabolic disorders. Anti-PD-L1 imaging enables direct detection of brown adipocytes independent of their metabolic activity. To verify this, we determined whether PD-L1 levels change in brown fat in response to signals that activate BAT. We first subjected C57BL/6 WT mice to a cold shock at 4 °C for 1 h, followed by imaging with ^64^Cu-labeled B3. Consistent with the hypothesis that PD-L1 expression is not substantially altered by BAT activation, cold-exposed mice showed PD-L1 signals that were identical to the signal from mice housed at room temperature (25 °C) (Fig. 4a, b, and Supplementary Fig. 4a), with a corresponding increase in Ucp-1 mRNA (Fig. 4c); no clear change in Ucp-1 protein was detectable over the time interval examined (Supplementary Fig. 4b). Whereas BAT is directly activated by signals through the β-adrenergic receptor, exposure of animals to the β-agonist CL-316243 led to minimal changes in PD-L1 expression compared to naive animals (Fig. 4d, e, and Supplementary Fig. 4c), despite a small increase in Ucp-1 (Fig. 4f). The addition of propranolol to CL-316243-treated animals also had no appreciable effect on PD-L1 signal intensity (Supplementary Fig. 4d). In addition, mice treated with CL-316243 showed a clear increase in ^18^F-FDG signal from BAT, while those same animals treated 1 day later with a second dose of CL-316243 showed no such increase in PD-L1 signal, leading to an increase in the ^18^F-FDG /PD-L1 signal ratio associated with β-agonist treatment (Fig. 4g–k; Supplementary Movies 2–5). Collectively, these findings demonstrate that PD-L1 is the first identified activation-independent marker of BAT suitable for live animal imaging.

## Discussion

The PD-1/PD-L1 axis has attracted attention in immunotherapy of cancer, mostly because of its correlation with exhaustion of T cells and the ability to reverse such exhaustion through application of blocking antibodies. We now show that BAT is the principal tissue expressing PD-L1 in the naive mouse, with signal intensities higher than from any other tissue and comparable to murine malignancies such as the widely used B16 melanoma. A variety of cell types express PD-L1 at low levels. Many normal and malignant tissues increase PD-L1 expression in response to IFN-γ. Previous studies have speculated that PD-L1 may be expressed on adipose tissue-associated hematopoietic cells as a marker of immune cell infiltration, based largely on low-resolution imaging and ex vivo scintillation counting. Through a combination of biochemical and genetic approaches we show that brown adipocytes themselves express PD-L1. Using high resolution VHH-based anti-PD-L1 immuno-PET, we identify even small brown adipose deposits within the mouse thorax, independent of strain or age. PD-L1 should thus be considered a general marker for brown fat in preclinical mouse models, where it can be used also for longitudinal observations. In humans, brown fat deposits are generally small, and are unlikely to be properly visualized using traditional antibody scaffold-based imaging, as is suggested by the comparatively poor resolution of imaging techniques that use full-sized antibodies in the mouse.

In live animals brown fat is imaged almost exclusively by using ^18^F-FDG, a glucose analog that is taken up by metabolically active cells, including brown adipocytes. However, relying on metabolic activity as the only means of detecting brown fat has obvious limitations, particularly when aiming for modification of its activity either genetically or pharmacologically. We found that PD-L1 expression was largely independent of metabolic activity and could be used to label BAT in naive mice as well as in animals undergoing cold stress or treatment with β-agonists. Our results establish the first available method to detect and measure total brown fat volume independent of its metabolic activity. Whether PD-L1 is also expressed on beige adipocytes in mice is presently unclear, but is an important area of investigation. Given the clinical interest in PD-L1 as both a therapeutic target and a tumor biomarker, whether this molecule is also present on beige and brown adipocytes in humans is a question that needs answering. The size of human brown fat stores may not allow their visualization using currently available full-sized ^89^Zr conjugated antibodies for imaging, due to the poor resolution of antibody-based PET. An approach similar to that described here may need to be developed to enable visualization of human PD-L1.

In addition to their well-studied role in modulating TCR signaling, clues that the PD-1/PD-L1 axis also affects metabolic pathways are beginning to emerge^30–32^. B16 melanoma expresses both PD-1 and PD-L1, with signaling through PD-1 on tumor cells leading to enhanced tumorigenesis. When PD-1 binds to its target ligand, T cells show repressed glycolysis and enhanced lipolysis^32^, with a possible role for the T cell-specific costimulatory molecule CD28, although details of the signaling events that control these effects remain to be worked out. Whether the findings reported for T cells represent a wider role for PD-1/PD-L1 in metabolic regulation remains unknown. Signaling properties of PD-L1 have gone mostly unexplored. Our data not only demonstrate a clear role for PD-L1 as a marker for brown fat, but also suggest the possibility that PD-L1 has a more general metabolic function, potentially even regulating the activity of this important metabolic organ.

## Methods

### Animals

All mice were housed at either the Dana Farber Cancer Institute or the Whitehead Institute for Biomedical Research and were maintained according to protocols approved by the DFCI Committee on Animal Care or the MIT Committee on Animal Care, respectively. C57BL/6 and BALB/c mice were purchased from Jackson Labs or bred in house. PD-L1 knockout mice were a gift from Arlene Sharpe (Harvard Medical School; Boston, MA). A three year old male alpaca (*Vicugna pacos*) was purchased locally, maintained in pasture, and immunized following a protocol authorized by the Tufts University Cummings Veterinary School Institutional Animal Care and Use Committee (IACUC).

### VHH Library generation

A male alpaca (*Vicugna pacos*) was immunized with a mixture of recombinant mouse and human proteins, including mouse PD-L1. A primary immunization was followed by four boosts, spaced two weeks apart, each containing 200 μg of mPD-L1 mixed 1:1 in alum (Thermo Scientific). Following immunization, total RNA was isolated from  ~ 10^6^ fresh PBLs using the RNeasy Plus Mini Kit (Qiagen), following the manufacturer’s instructions. First strand cDNA synthesis was performed using SuperScript III reverse transcriptase (Life Technologies) and a combination of oligo dT, random hexamer or immunoglobulin-specific primers, AlCH2 and AlCH2.2, as previously described^22^. PCR amplification of VHH sequences and phage library generation followed a previously published procedure. Following transformation into TGI cells (Agilent), the total number of independent clones was estimated to be 3.2 × 10^6^. Ninety-six clones were selected at random and sequenced to assess library diversity. The resulting phagemid libraries were stored at −80 °C.

### Selection of VHHs by phage display

Two hundred microliters of the immunized VHH phage library was inoculated in 100 ml super optimal broth (SOC) with 50 µg/ml ampicillin. The culture was grown to mid-log phase, and infected with 100 µl 10^14^ PFU/ml VCSM13 helper phage. The culture was then incubated for 2 h at 37 °C, the cells collected by centrifugation, and re-suspended in 100 ml 2YT, 0.1% glucose, 50 µg/ml kanamycin and 50 µg/ml ampicillin. Cultures were incubated overnight at 30 °C, then centrifuged for 20 min at 7700 × g, followed by phage precipitation from the resulting supernatant with 20% PEG-6000/500 mM NaCl at 4 °C, and resuspension in PBS.

One hundred micrograms of recombinant mouse PD-L1-Fc was biotinylated by coupling Chromalink NHS-biotin reagent (Solulink) to primary amines for 90 min in 100 mM phosphate buffer, pH 7.4, 150 mM NaCl. The reaction was then run through an Amicon 10 kDa MWCO concentrator (EMD Millipore) to remove remaining NHS-biotin. Incorporation of biotin was monitored spectrophotometrically, following the manufacturer’s guidelines. 100 µl MyOne Streptavidin-T1 Dynabeads (Life Technologies) were blocked in PBS plus 2% (w/v) mouse serum in a microfuge tube for 2 h at 37 °C. Following blocking, 20 µg biotinlyated PD-L1-Fc in mouse serum was added to the beads, and incubated for 30 min at room temperature, with agitation. The beads were then washed 3× in PBS, and 200 µl of 1014 PFU/ml M13 phage displaying the VHH library were added in PBS/mouse serum for 1 h at room temperature. The beads were then washed 15× with PBS, 0.1% Tween-20. Phage was eluted by the addition of *E. coli* ER2738 (NEB) for 15 min at 37 °C, followed by elution with 200 mM glycine, pH 2.2, for 10 min at room temperature. The glycine elution was neutralized and pooled with the *E. coli* ER2738 culture, and plated onto 2YT agar plates supplemented with 2% glucose, 5 µg/ml tetracycline and 10 µg/ml ampicillin, and grown overnight at 37 °C. A second round of panning was performed with the following modifications: 2 µg of biotinylated PD-L1-Fc was used as bait, and incubated with 2 µl 1014 PFU/ml M13 phage displaying the first-round VHH library for 15 min at 37 °C, followed by extended washes in PBS with 0.1% Tween-20.

Following two rounds of phage panning, 96 colonies were isolated in 96-well round-bottom plates and grown to mid-log phase at 37 °C in 200 µl 2YT, 10 µg/ml ampicillin, 5 µg/ml tetracycline, induced with 3 mM IPTG and grown overnight at 30 °C. Plates were centrifuged at 12 X kg for 10 min, and 100 µl of supernatant was mixed with an equal volume of PBS, 5% (w/v) nonfat dry milk. This mixture was added to an ELISA plate coated with 1 µg/ml PD-L1-Fc. Following four washes in PBS, 1% Tween-20, anti-Etag antibody (Bethyl) was added at a 1:10,000 dilution in PBS, 5% (w/v) nonfat dry milk for 1 h at room temperature. The plate was developed with fast kinetic TMB (Sigma) and quenched with 1 M HCl. Absorbance at 450 nm was determined in a plate reader (Spectramax, Molecular Devices).

### Cloning and expression of B3 and A12

B3 coding sequence were sub-cloned into the *E. coli* periplasmic expression vector pHEN6 using the PCR primers 189 (5′-tactcgcggcccagccGGCCCAACCGGCCATGGC-3′) and 190 (5′-agtcctcctgaggagacggtgaccGAGACGGTGACCTGGGTCCCC-3′) to allow for Gibson cloning and the inclusion of a C-terminal sortase motif and 6xHis tag. WK6 *E. coli* containing the plasmid were grown to mid-log phase at 37 °C in TB plus ampicillin, and induced with 1 mM IPTG overnight at 30 °C. Cells were harvested by centrifugation at 5000 × *g* for 15 min at 4 °C, then resuspened in 25 ml 1x TES buffer (200 mM Tris, pH 8, 0.65 mM EDTA, 0.5 M sucrose) per liter culture, and incubated for 1 h at 4 °C with agitation. Resuspended cells wre then submitted to osmotic shock by diluting 1:4 in 0.25× TES, and incubating overnight at 4 °C. The periplasmic fraction was isolated by centrifugation at 8000 rpm for 30 min at 4 °C, and then loaded onto Ni-NTA (Qiagen) in 50 mM Tris, pH 8, 150 mM NaCl and 10 mM imidazole. Protein was eluted in 50 mM Tris, pH 8, 150 mM NaCl, 500 mM imidazole and 10% glycerol, then loaded onto a Superdex 75 10/300 column in 50 mM Tris, pH 8, 150 mM NaCl, 10% glycerol. Recombinant VHH purity was assessed by SDS-PAGE, and peak fractions were recovered and concentrated with an Amicon 10,000 KDa MWCO filtration unit (Millipore), and stored at −80 °C.

### C-terminal labeling of VHHs with Biotin or Alexa647

A heptamutant variant of *S. aureus* Sortase A was used to label B3 and A12 by incubating 30 uM of purified VHH protein with 5uM 7 M SrtA and 100 uM GGGK-Biotin or GGGK-Alexa647 nucleophiles in 50 mM Tris, pH 8, 150 mM NaCl for 2 h at room temperature. Unreacted VHH and 7 M SrtA were removed by adsorption onto Ni-NTA agarose beads (Qiagen). The unbound fraction was concentrated and excess nucleophile with an Amicon 3,000 KDa MWCO filtration unit (Millipore), and stored at −80 °C.

### ELISAs

High binding plates microtiter plates (Corning) were coated with recombinant Fc fusion proteins (S.A.) overnight at 25 ng/ml (CTLA-4, mPD-L1) or 100 ng/ml (hPD-L1) in carbonate buffer. Biotinylated VHHs or antibodies (BD Biosciences) were incubated on the coated plates in 10% inactivated fetal calf serum in phosphate buffered saline for 1 h in the presence or absence of unlabeled VHH or antibody across a range of concentrations, washed in 0.5% Tween in PBS and then developed using streptavidin-HRP (BD biosciences) and tetramethylbenzidine (TMB). For plate-bound ligand binding assays, B7-1 and PD-1 Fc fusions (R&D systems) were incubated with plate-bound ligands for 1 h in the presence of VHHs or antibodies. Plates were then washed and binding was determined using biotinylated polyclonal antibodies against B7-1 and PD-1 (R&D systems, 1:1000 dilution) respectively, followed by incubation with streptavidin-HRP and then development with TMB.

### Flow cytometry

Cells were analyzed using a FACS Fortessa (BD) or LSR II (BD). All antibodies were directly conjugated and obtained from BD Pharmingen with the exception of antibodies to mouse and human PD-L1 (BioLegend), mouse CTLA-4 (BioLegend), mouse TIM3 (BioLegend) and mouse CD44 (Ebiosciences). All antibodies were used at a 1:100 dilution. Staining protocols followed manufacturers instructions. VHHs used in flow cytometry were directly conjugated to Alexa647, diluted to 2 µg/ml in 10% IFCS in PBS and incubated with cells for 15 min on ice. Spleen populations were identified using the following markers: B cells (CD19 + ), CD4 T cells (CD3 + CD4 + ), CD8 T cells (CD3 + CD8 + ), myeloid DCs (CD19-CD3-CD11b + CD11c + ) according to manufacturers’ recommendations (Supplmentary Figure 6).

### Bone marrow chimera generation

Donor bone marrow was harvested from the femurs of WT or PD-L1 KO mice. Recipients were irradiated with 600 rads at time 0 and 4 h for a total of 1200 rads. One hour later, they were injected by tail vein with 100 μl collected bone marrow cells in PBS. Two donor animals were used for each recipient with ~ 5 × 106 bone marrow cells used per transfusion. After transfusion, mice were housed in autoclaved cages, and were treated with Septra water for 2 weeks. Imaging analysis was performed 6-7 weeks after bone marrow transfusion.

### In vitro differentiation of BAT

BAT was isolated and cultured following previously described methods^27^. Briefly, 2 to 3-week-old C57BL/6, PD-L1 KO or PD-1 mice were sacrificed, and interscapular BAT was collected, minced and digested with and with collagenase digestion buffer (Hanks balanced salt solution, 0.2% collagenase, 2% BSA). Pre-adipocytes cells were collected by sequential filtering through 70 µm and 40 µm membranes and centrifugation. Pre-adipocytes were cultured to confluence in DMEM supplemented with glutamine (Life Technologies), 5% new-born calf serum (Invitrogen), 5% fetal calf serum and pen/strep (Life Technologies), and induced to differentiate for 5 days with DMEM containing 10% fetal bovine serum (Life Technologies), 850 nM insulin (Sigma), 0.5 µM dexamethasone (Sigma), 250 µM 3-isobutyl-1-methylxanthine, phosphodiesterase inhibitor (IBMX, Sigma), 1 µM rosiglitazone (Sigma), 1 nM T3 (Sigma).

### Brown adipocyte isolation for flow cytometry

Mice were euthanized and BAT was surgically removed from the interscapular region of BL6 WT or PD-L1 KO mice and associated WAT was removed. Tissue was minced with scissors and then digested for 30 min at 37 C with vigorous shaking (350 rpm). After filtration (150 um mesh) and dilution in room temperature DMEM, the cell mixture was centrifuged (700 g) at room temperature. Mature brown adipocytes (floating fraction) separated from the stromal vascular fraction (SVF) on top of the solution and were carefully pipetted off using a 1 ml pipette tip with the tip cut off to allow aspiration of larger pieces. The SVF fraction at the bottom of the tube was separately analyzed and is the source of the CD45 + cells (this is unclear). Cells were stained for 10 min at room temperature with either isotype control antibody or anti-PD-L1 (10 F.9G2), washed, and resuspended after centrifugation. Both fractions were analyzed by flow cytometry. Brown adipocytes were identified by size and granularity, and by negative staining for CD45 as previously described. Cells were analyzed on an LSRFortessa (BD Biosciences).

### Quantitative PCR

Total RNA was isolated from in vitro cultured cells at different time points of differentiation using TRizol reagent (Life Technologies/Ambion) and a miRNeasy Mini Kit (Qiagen). Three hundred nanograms were reverse transcribed using Superscript II reverse transcriptase (LifeTechnologies/Invitrogen) using 300 ng random hexamers (LifeTechnologies/Invitrogen). The cDNA was diluted 1:10 and 2.5 µl for a 96-well plate or 1 µl for a 384 well plate were used for quantitative Real-time PCR. qPCR was carried out on an ABI7900HT Fast real-time PCR system (Applied Biosystems) and analyzed using the delta delta Ct method normalized to 18S. The following primer sets were used: 18S fwd 5′-GTAACCCGTTGAACCCCATT-3′; 18S rev 5′-CCATCCAATCGGTAGTAGC-3′; Ucp1 fwd 5′-ACTGCCACACCTCCAGTCATT-3′;

Ucp1 rev 5′-CTTTGCCTCACTCAGGATTGG-3′; Pd-l1 fwd 5′-TGCTGCATAATCAGCTACGG-3′; Pd-l1 rev 5′-CCACGGAAATTCTCTGGTTG-3′.

### Fluorescence microscopy

Adult WT mice were injected IP with 50 ug of B3-Alexa647 or 96G3m-Alexa647 (control VHH). Mice were sacrificed one hour after injection and interscapular BAT was collected, mounted on a coverslip and imaged with a Zeiss AxioPlan 2 microscope. Images were analyzed using Metamorph and ImageJ software.

### Imaging

To label purified VHH with tetrazine, reactions were performed in 50 mM Tris·HCl, pH 7.5 supplemented with 10 mM CaCl2, 150 mM NaCl, 500uM triglycine-containing probe, 100 μM VHH and 5 μM sortase. After incubation at 4 °C with agitation for 2 h, reaction products were analyzed by LC-MS, with yields generally > 90%. Ni-NTA beads were added to the reaction mixture with agitation for 5 min at 25 °C, followed by centrifugation to remove sortase and any remaining unreacted His-tagged substrate. The final product was purified by size-exclusion chromatography in PBS. VHH-Tz (40 μl, 150 μM), PBS (300 μl), and 18F-TCO in DMSO (4.0 mCi (148.0 MBq), 100 μl) were mixed in a microfuge tube. The tube was sealed and shaken at room temperature for 20 min. The mixture was analyzed by radio-TLC (instant thin layer chromatography (ITLC), 100% MeCN, Rf 18F-TCO = 0.9, Rf 18F-VHH = 0.0) showing 90% conversion to 18F-VHH. The reaction mixture was loaded onto a PD-10 size-exclusion cartridge (GE Healthcare), and eluted with PBS. Imaging was performed on a Siemens Inveon PET/CT imager. All PET/CT imaging procedures were approved by the Massachusetts General Hospital subcommittee on research animal care.

### Murine PD-L1 antigen production in HEK 293 cells

Coding sequences were cloned into a modified version of the Daedalus lentiviral transfer vector, which supported ligation independent cloning and expressed targets as a fusion with a variant of mIgG2a. Lentivirus production was essentially as described. Transduction of HEK-293F cells (Gibco) was carried out at a scale of 30 ml in Freestyle 293 Expression media (Gibco) and cultures were expanded to a 3 l production scale. Supernatant from 3 l culture was harvested by centrifugation (2000 × *g*, 10 min), 15 ml 1 M MES pH 6.5 and AEBSF (final concentration 20 µM) was added, and the supernatant swirled at 4˚C for 1 h with a 30 ml bed volume (bv) of His60 Ni-IDA resin (Clontech). Resin was poured into a gravity column, washed with 10 bv wash buffer (25 mM MES, pH 6.5, 150 mM NaCl, 10% glycerol, 50 mM Arginine-Cl and 5 mM imidazole)) and eluted with 5bv elution buffer (wash buffer with 100 mM Arginine-Cl and 0.5 M imidazole). Protein was concentrated using centrifugal units (Amicon Ultracel-10, Millipore), and applied to a gel filtration column (Sephacryl S200 26/60, GE lifesciences) equilibrated in 25 mM MES, pH 6.5, 150 mM NaCl, 10% glycerol, 100 mM Arg-Cl. Peak fractions were collected and concentrated by as above.

### Murine PD-L1 site-specific mutagenesis

Murine PD-L1 was cloned into the SacI and BamHI sites of the Clontech N1 mCherry-fusion vector replacing the native leader peptide sequence with that of erythropoietin (EPO). Site-specific mutagenesis was performed using KOD polymerase, 2 mM dNTPs and 4 mM MgCl2. Solvent accessible positions for mutagenesis were selected based on the crystal structure of the complex formed by human PD-L1 and PD-1 (PDB: 3BIK) and determining the equivalent positions in mouse PD-L1 using Custal2 sequence alignment. Each chosen position was mutated to an Ala, Glu or Arg residue with an overall success rate of  ~ 70%. All sequence-validated mutants were tested by transient expression in suspension HEK 293 cells prior to use in the nanobody epitope mapping experiments.

### B3 epitope mapping

Wild-type and mutant PD-L1 mCherry-fusion constructs were transiently transfected into HEK 293 S cells plated in 24-well tissue culture plates using 1 ml of Freestyle media (Invitrogen) and PEI transfection. Three days post transfection, 12 wells were selected at random and counted, and the average count used to dilute cells to ~ 1 × 106 cells/ml in PBS. The Alexa 488-labeled α-PD-L1 VHH was diluted to 0.01 μg/μl in 1X PBS and 0.2% BSA. Binding reactions were setup in 96-well V-bottom plates using 150 μl cells (150,000) and 50 μl of diluted nanobody. Reactions were covered in foil and incubated at room temperature with shaking at 900 rpm in a 96-well plate shaker for 1 h. After incubation, plates were centrifuged at 500 × *g* for 5 min, the supernantant removed and 200 μl of 1× PBS and 0.2% BSA was added to wash. After two additional washes, cells were resuspended in 100 μl and analyzed using an Hypercyte autosampler (Intellicyt) connected to a custom Blue-Yellow laser BD Accuri flow cytometer. Counts were normalized to wild type.

### Statistics

Two sample comparisons used the *t*-test with pooled variance if there was no evidence of inhomogeneity of variances between groups. If the variances were unequal, the exact Wilcoxon rank sum test, a non-parametric alternative to the *t*-test, was used. Every effort was made to keep testing consistent across related experiments. For comparisons of more than two groups, analysis of variance (ANOVA) was used if there was no evidence of inhomogeneity of variance; the Kruskal-Wallis test was the non-parametric alternative. Tumor growth studies were analyzed using mixed model ANOVA. Sample size was determined based on a review of similar experiments in the literature or based on prior experience in the lab. Animals were all randomized to treatment groups and the start of the experiment. The experiments were not blinded unless stated in the methods.

### Data availability

The data sets generated and analyzed during current study are available from the corresponding author on reasonable request.

## Electronic supplementary material

Supplementary Information

Description of Additional Supplementary Files

Supplementary Movie 1

Supplementary Movie 2

Supplementary Movie 3

Supplementary Movie 4

Supplementary Movie 5
