# Supplementary material for: PD-L1 is an activation-independent marker of brown adipocytes
Source: Nat Commun. 2017 Sep 21;8:647. doi: 10.1038/s41467-017-00799-8 (PMC5608754; doi:10.1038/s41467-017-00799-8)
Supplement: Supplementary file 1 — Supplementary Information [file 41467_2017_799_MOESM1_ESM.pdf]

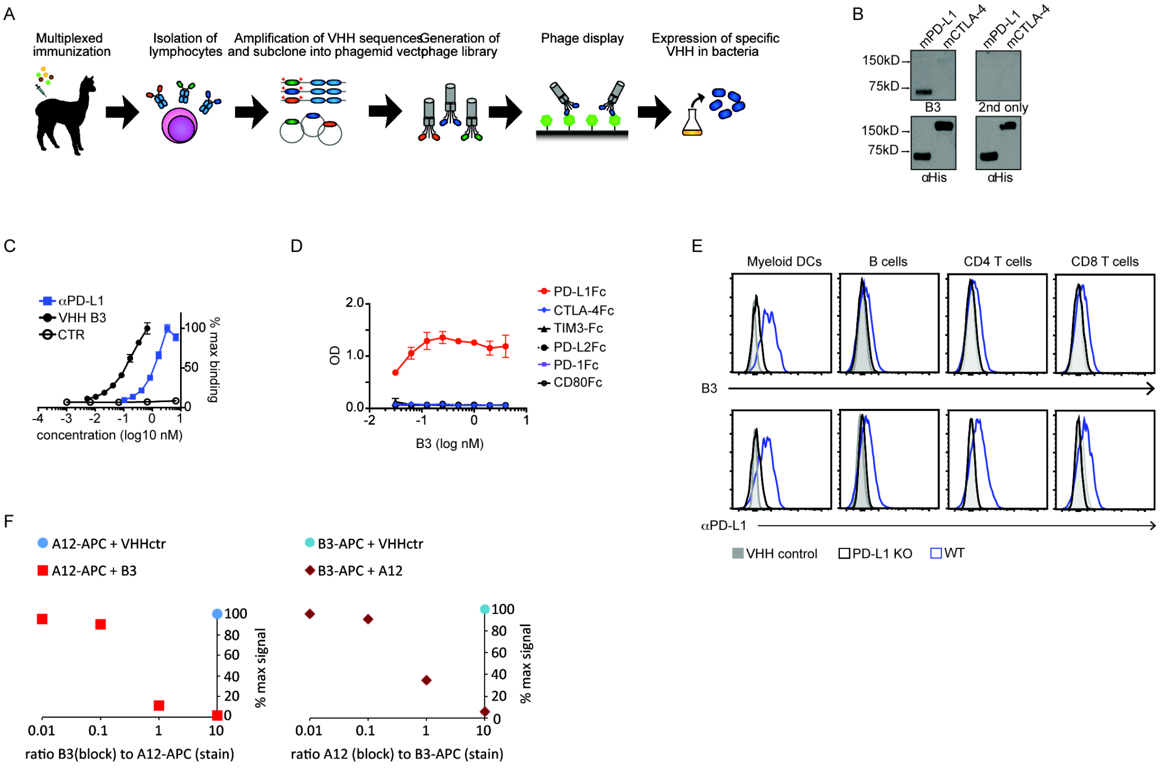

Supplementary Figure 1. VHH B3 recognizes PD-L1.
(A) Schematic showing immunization and isolation of candidate PD-L1 reactive VHHs. (B) VHH immunoblot against murine (m)PD-L1 and mCTLA-4. (C) Biotinylated VHH or antibody was incubated with plate bound mPD-L1-Fc. Binding was detected using streptavidin-HRP and TMB. Data are normalized to the maximum signal. Error bars show standard deviation (SD). (D) Biotinylated B3 was incubated with plate bound Ig superfamily members as indicated, and binding was assessed as in (C). (E) Flow cytometry on splenic populations as indicated from WT and PD-L1 KO mice using VHH B3 or αPD-L1 antibody. (F) IFN-g treated B16 cells were stained with APC conjugated B3 or A12 as indicated (stain) in the presence of unconjugated B3, A12, or VHHctr (block) as indicated, and analyzed by flow cytometry. Graphs show percentage of maximum APC signal as measured by mean fluorescence intensity.

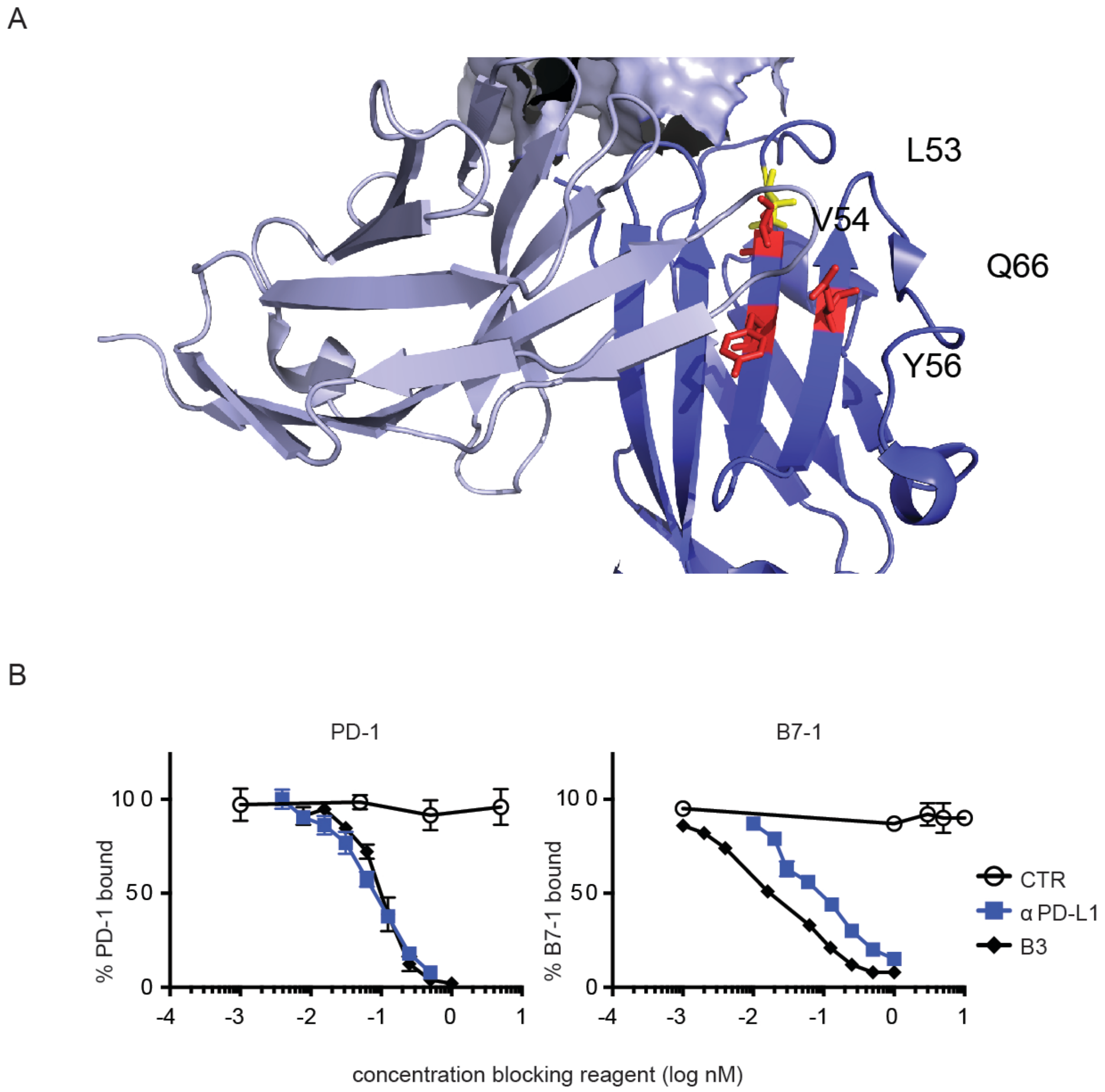

Supplementary Figure 2. VHH B3 blocks binding of PD-L1 to its ligands.
(A) PD-L1 mutants with reduced B3 binding mapped onto the crystal structure of the PD-L1/PD-1 complex. Red residues = strong reduction in binding; yellow = weak reduction. (B) PD-1-Fc (left) or B7-1-Fc (right) were incubated with plate bound PD-L1-Fc in the presence of VHH or antibody as indicated. Binding was detected using biotinylated polyclonal antibodies against B7-1 and PD-1 developed using streptavidin-HRP and TMB. Data are normalized to the maximum signal. Error bars show standard deviation (SD).

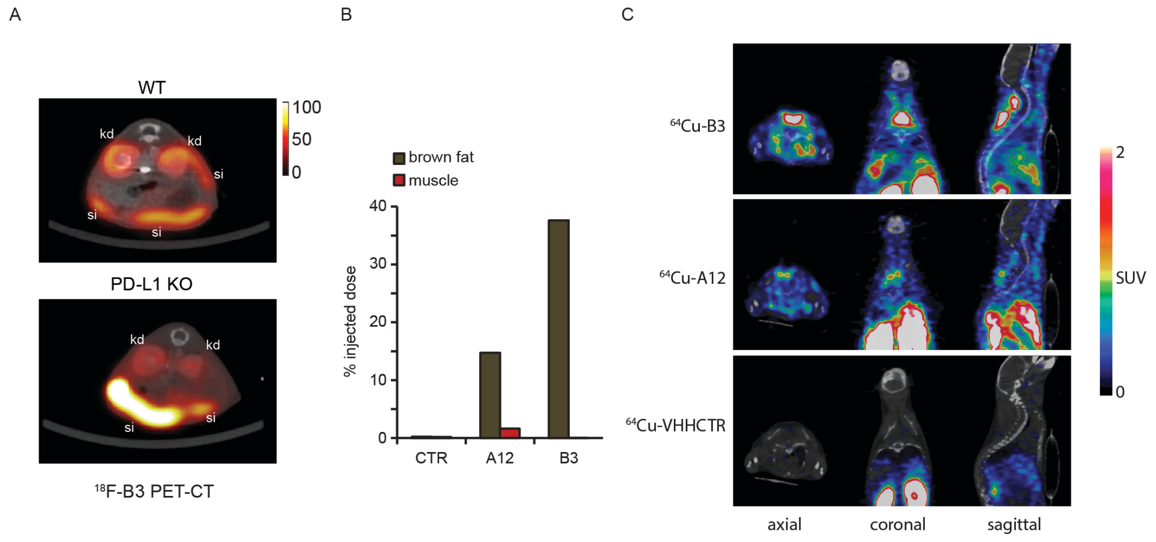

Supplementary Figure 3. VHHs against PD-L1 localize to BAT.
(A) cross-sectional axial PET-CT images from WT (top) and PD-L1 KO (bottom) mice imaged with ^{18}F-B3. The images clearly demonstrate staining within the lumen of the small intestine (si) and kidney (kd). (B) Scintillation counts from mice injected with ^{64}Cu-VHH conjugates as indicated comparing extracted anatomical BAT to muscle. (C) PET-CT image slices through the BAT from the mice analyzed in (B).

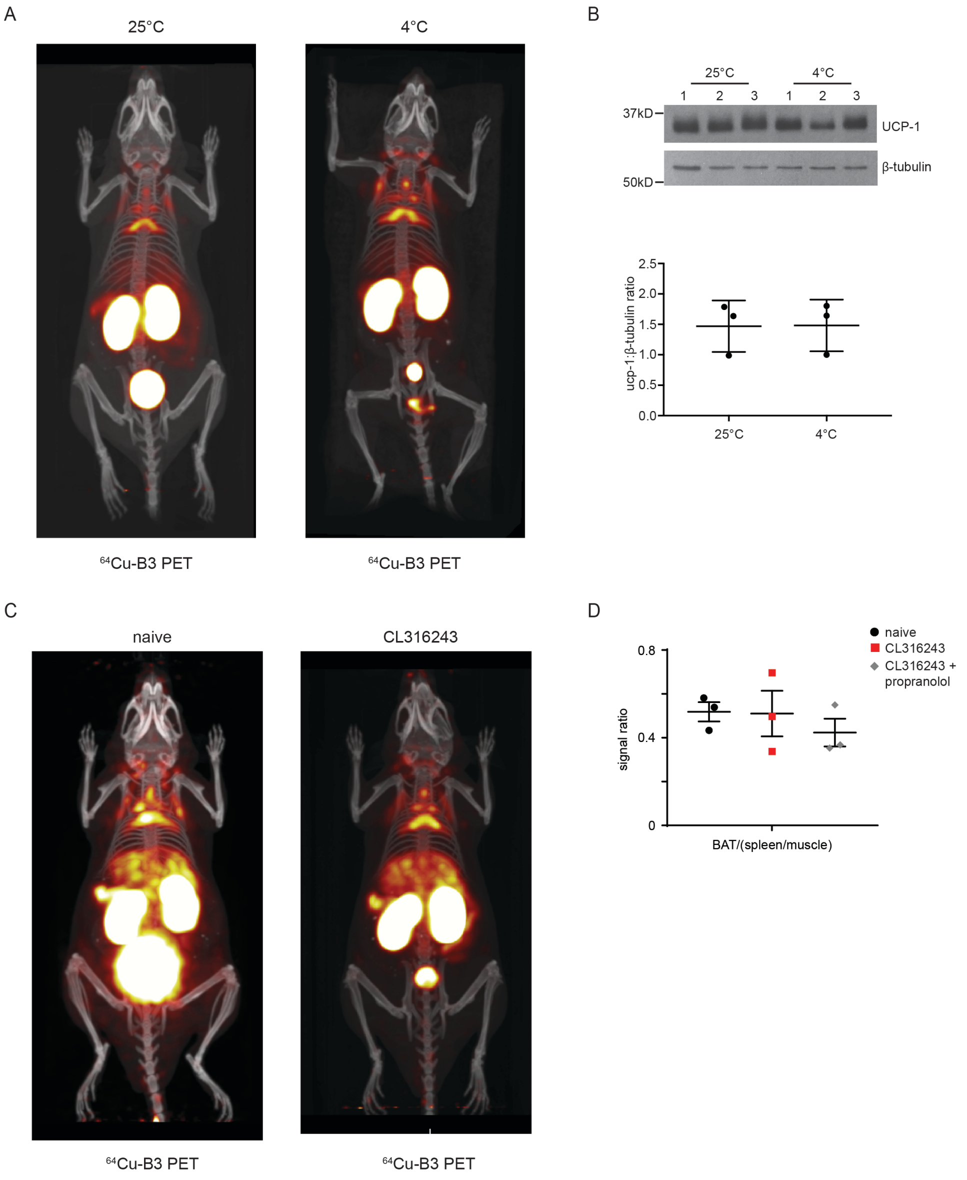

Supplementary Figure 4. The beta-blocker propranolol does not alter PD-L1 expression in beta-agonist treated mice.
(A) and (C) Full body 3D projection PET-CT images of the animals pictured in Figure 4. (A) room temperature (left panel) and 4°C exposed (right panel) C57BL/6 WT mice. (B) Immunoblot for UCP-1 (top panel) on lysates from BAT excised from the mice included in (A) with quantification of normalized UCP-1 signal intensity (bottom panel). (C) naïve (left panel) and CL316243 treated (right panel) mice. (D) Signal ratio of BAT normalized to spleen and muscle from the mice in Figure 4E alongside parallel imaged mice pre-treated with 15 mg/kg propranolol followed 30 minutes later by CL316243. All error bars show SEM (standard error of mean).

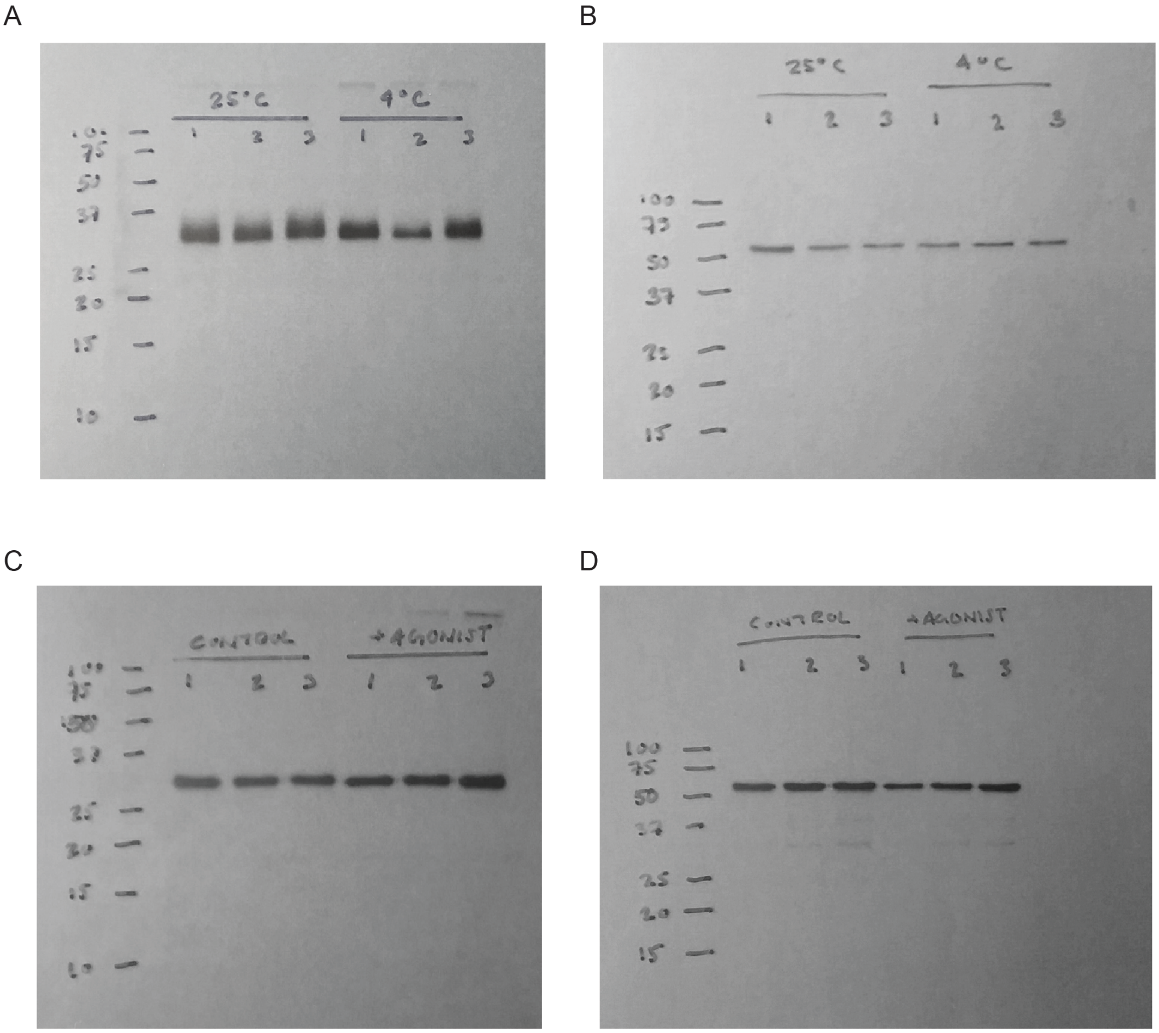

Supplementary Figure 5. Full-sized immunoblots.
(A) Immunoblot for UCP-1 on lystates from BAT excised from the C57BL/6 WT mice at room temperature and at 4°C. (B) Immunoblot for beta-tubulin on lystates from BAT excised from the C57BL/6 WT mice at room temperature and at 4°C. Cropped versions of blots are present in Supplementary Figure 4.(C) Immunoblot for UCP-1 on lystates from BAT excised from naive C57BL/6 WT mice and C57BL/6 WT mice treated with CL316243. (D) Immunoblot for UCP-1 on lystates from BAT excised from naive C57BL/6 WT mice and C57BL/6 WT mice treated with CL316243. Cropped versions of blots are present in Figure 4. In all panels, numbers on left indicated molecular weights in kDa of protein standards. Numbers at tops of panels indicate biological replicates.

Basic Schematic Illustrating Immune Cell Gating

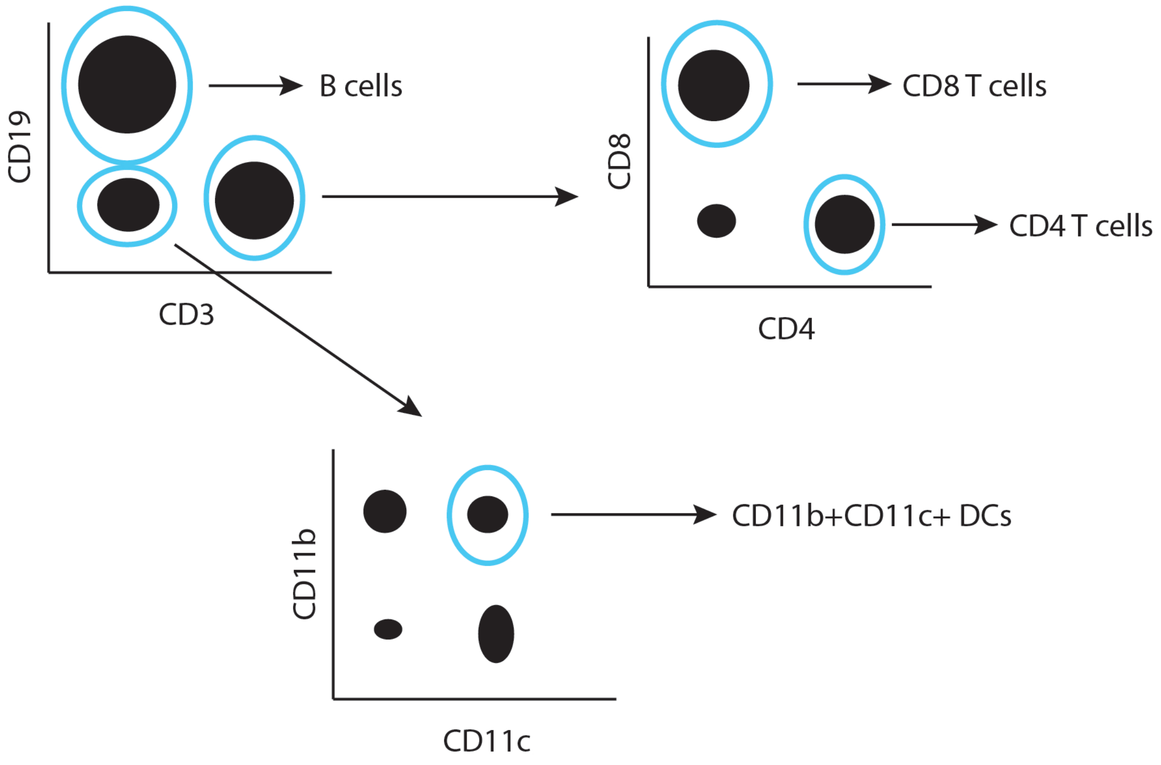

Supplementary Figure 6.
Basic schematic illustrating immune cell gating strategy using standard commercially available antibodies as indicated. This strategy was used for spleen cells, and for CD45+ cells harvested from the BAT.
