# Supplementary material for: PD-L1 is an activation-independent marker of brown adipocytes
Source: Nat Commun. 2017 Sep 21;8:647. doi: 10.1038/s41467-017-00799-8 (PMC5608754; doi:10.1038/s41467-017-00799-8)
Supplement: Supplementary file 2 — Description of Additional Supplementary Files [file 41467_2017_799_MOESM2_ESM.pdf]

Description of Additional Supplementary Files

File Name: Supplementary Movie 1
Description: This 3D representation corresponds to the still image presented in Figure 1J: ^{18}F- B3 image from a WT mouse injected with subcutaneous B16 melanoma.

File Name: Supplementary Movie 2
Description: ^{18}F-FDG PET-CT 3D projection image acquired as on naïve C57BL/6 mouse.

File Name: Supplementary Movie 3
Description: ^{18}F-FDG PET-CT 3D projection image acquired as on a C57BL/6 mice treated with 10 mg/kg of the β-adrenergic agonist CL316243 given i.p. 45 minutes before injection of radiolabel.

File Name: Supplementary Movie 4
Description: ^{64}Cu-B3 PET-CT 3D projection image acquired as on naïve C57BL/6 mouse.

File Name: Supplementary Movie 5
Description: ^{64}Cu-B3 PET-CT 3D projection image acquired as on a C57BL/6 mice treated with 10 mg/kg of the β-adrenergic agonist CL316243 given i.p. 45 minutes before injection of radiolabel.
